# Artificial intelligence-assisted metastasis and prognosis model for patients with nodular melanoma

**DOI:** 10.1371/journal.pone.0305468

**Published:** 2024-08-07

**Authors:** Chan Xu, Xiaoyu Yu, Zhendong Ding, Caixia Fang, Murong Gao, Wencai Liu, Xiaozhu Liu, Chengliang Yin, Renjun Gu, Lu Liu, Wenle Li, Shi-Nan Wu, Bei Cao

**Affiliations:** 1 State Key Laboratory of Molecular Vaccinology and Molecular Diagnostics & Center for Molecular Imaging and Translational Medicine, School of Public Health, Xiamen University, Xiamen, China; 2 Department of Oncology, Taicang TCM Hospital Affiliated to Nanjing University of Chinese Medicine, Suzhou, China; 3 Department of Anesthesiology, The Third Affiliated Hospital of Sun Yat-sen University, Guangzhou, China; 4 Department of Pharmacy, Qingyang City People’s Hospital, Qingyang, China; 5 Beijing Rehabilitation Hospital Affiliated to Capital Medical University, Beijing, China; 6 Department of Orthopaedics, Shanghai Jiao Tong University Affiliated Sixth People’s Hospital, Shanghai, China; 7 Department of Cardiology, The Second Affiliated Hospital of Chongqing Medical University, Chongqing, China; 8 Faculty of Medicine, Macau University of Science and Technology, Macau, China; 9 School of Chinese Medicine & School of Integrated Chinese and Western Medicine, Nanjing, University of Chinese Medicine, Nanjing, China; 10 Department of Dermatology, the First Affiliated Hospital of Anhui Medical University, Hefei, Anhui, China; 11 Institute of Dermatology, Anhui Medical University, Hefei, Anhui, China; 12 Eye Institute of Xiamen University, School of Medicine, Xiamen University, Xiamen, Fujian, China; 13 Department of Thyroid Surgery, The Affiliated Hospital, Southwest Medical University, Luzhou, China; University of Wisconsin-Eau Claire, UNITED STATES

## Abstract

**Objective:**

The objective of this study was to identify the risk factors that influence metastasis and prognosis in patients with nodular melanoma (NM), as well as to develop and validate a prognostic model using artificial intelligence (AI) algorithms.

**Methods:**

The Surveillance, Epidemiology, and End Results (SEER) database was queried for 4,727 patients with NM based on the inclusion/exclusion criteria. Their clinicopathological characteristics were retrospectively reviewed, and logistic regression analysis was utilized to identify risk factors for metastasis. This was followed by employing Multilayer Perceptron (MLP), Adaptive Boosting (AB), Bagging (BAG), logistic regression (LR), Gradient Boosting Machine (GBM), and eXtreme Gradient Boosting (XGB) algorithms to develop metastasis models. The performance of the six models was evaluated and compared, leading to the selection and visualization of the optimal model. Through integrating the prognostic factors of Cox regression analysis with the optimal models, the prognostic prediction model was constructed, validated, and assessed.

**Results:**

Logistic regression analyses identified that marital status, gender, primary site, surgery, radiation, chemotherapy, system management, and N stage were all independent risk factors for NM metastasis. MLP emerged as the optimal model among the six models (AUC = 0.932, F1 = 0.855, Accuracy = 0.856, Sensitivity = 0.878), and the corresponding network calculator (https://shimunana-nm-distant-m-nm-m-distant-8z8k54.streamlit.app/) was developed. The following were examined as independent prognostic factors: MLP, age, marital status, sequence number, laterality, surgery, radiation, chemotherapy, system management, T stage, and N stage. System management and surgery emerged as protective factors (HR < 1). To predict 1-, 3-, and 5-year overall survival (OS), a nomogram was created. The validation results demonstrated that the model exhibited good discrimination and consistency, as well as high clinical usefulness.

**Conclusion:**

The developed prediction model more effectively reflects the prognosis of patients with NM and differentiates between the risk level of patients, serving as a useful supplement to the classical American Joint Committee on Cancer (AJCC) staging system and offering a reference for clinically stratified individualized treatment and prognosis prediction. Furthermore, the model enables clinicians to quantify the risk of metastasis in NM patients, assess patient survival, and administer precise treatments.

## Introduction

Cutaneous melanoma (CM), originating from cutaneous melanocytes, exhibits invasive growth and is distinguished by its high rates of metastasis and recurrence [1–3]. Nodular melanoma (NM) comprises approximately 14% of all CM cases and displays a higher mortality rate than other subtypes [4–6], with research indicating that it can be fatal in over 40% of melanoma patients [7].

The early diagnosis and identification of metastases are crucial for improving the prognosis and reducing mortality in NM. However, NM exhibits rapid growth and early invasiveness, with infiltration rates estimated at up to 0.5 mm/month, offering a narrower window for early diagnosis compared to other melanomas [8]. Moreover, NM deviates from the typical melanoma growth pattern, and its clinical presentation often lacks the classic features of melanoma, at times mimicking benign lesions that manifest as pink or mottled papules, thereby complicating early detection and diagnosis for both physicians and patients [9]. However, current conventional screening methods offer limited assistance in the early diagnosis and identification of metastases in NM. NM can present a nonspecific pattern on dermoscopy and lack identifiable melanoma features, potentially evading clinical and dermoscopic detection [10].

Beyond early diagnosis and metastases identification, comprehending the principal factors influencing disease progression and effectively and precisely assessing patient prognosis are imperative in determining optimal treatment strategies for patients. Currently, the American Joint Committee on Cancer (AJCC) staging system stands as the most prevalent method for assessing the prognosis of patients with NM [11, 12]. However, the AJCC system primarily includes information on the the tumor’s original site and distant metastases, omitting crucial details like patient-specific factors, additional tumor characteristics and treatment methods, thus limiting its predictive accuracy for CM patient prognosis [13, 14]. Prognostic nomograms forecast clinical outcome by integrating various prognostic variables into quantifiable values, presented visually, offering benefits over conventional TNM staging [15]. Consequently, some researchers have suggested it as an alternative to the AJCC staging system, potentially establishing a new prognostic benchmark.

Artificial intelligence (AI), underpinned by machine learning (ML) technologies, is currently advancing in the medical field and beyond [16–19]. AI can facilitate the modeling and prediction of medical data. Currently, research into the metastasis and prognosis of NM patients remains scant, with a notable absence of pertinent predictive models. It merits emphasis that a model that integrates ML algorithms for predict metastasis and prognosis in NM patients represents a pioneering endeavor.

This study’s objective is to identify patients with NM within the Surveillance, Epidemiology and End Result (SEER) database, to explore the prognostic factors associated with NM patients, and to integrate ML algorithms for predicting metastasis in NM patients, furthermore, to develop a nomogram for predicting the prognosis of NM patients based on these prognostic factors, and to validate and assess its efficacy. Additionally, a risk stratification system, derived from the nomogram scores, was created to classify NM patients into low-risk and high-risk categories. This approach will facilitate accurate and personalized prognostic assessment for NM patients, thereby aiding clinicians in tailoring treatment and follow-up strategies.

## Methods

### 1. Patient screening

**Fig 1:**
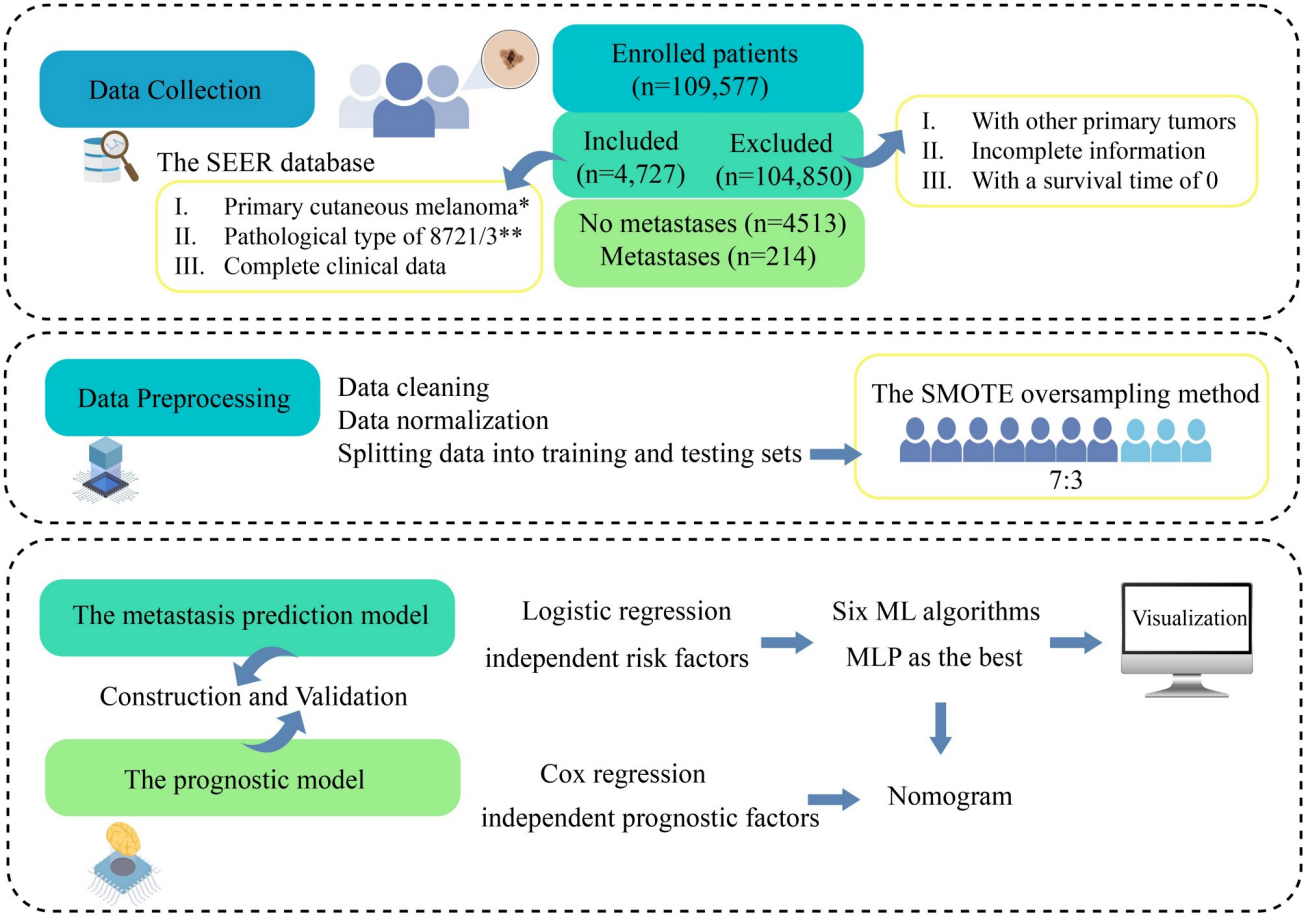
Overall research process. *According to the 3rd edition of the International Classification of Tumors code (Site recode ICD-O-3/WHO 2008). **8721/3 represents nodular melanoma. ML,Machine Learning; MLP, Multilayer Perceptron.

Clinical details for 4,727 NM patients were extracted from the SEER database, covering CM cases diagnosed between 2010 and 2015, in accordance with specified inclusion and exclusion criteria (Fig 1). Simultaneously, two independent collectors performed the extraction, with any disagreements resolved by a third arbitrator. SEER database information is anonymous, ensuring no breach of patient privacy. Furthermore, the SEER database is publicly accessible, negating the need for patient-informed consent.

### 2. Variables included in the study

Variables encompassed age, gender, race, marital status, sequence number, laterality, grade, primary site, mode of surgery at the primary site, AJCC 7th edition TNM staging, and radiotherapy information. X-tile software, utilizing Kaplan-Meier survival curves, classified patients into two age groups using with an optimal cutoff at 60 years, transforming continuous variables into categorical ones. Additionally, follow-up variables comprised survival months and vital status recodes (with study cutoff applied). The primary endpoint observed was overall survival (OS), defined as the duration from diagnosis to any-cause death by the end of follow-up.

### 3. Construction and validation of the metastasis prediction model

Independent risk factors for NM metastasis were identified through univariate and multivariate logistic regression analysis. To mitigate the impact of unbalanced data on the model construction, the SMOTE oversampling method was employed for preprocessing, with the oversampling data divided into a training set and a test set in a 7:3 ratio [20]. Six ML algorithms—Multilayer Perceptron (MLP), Adaptive Boosting (AB), Bagging (BAG), logistic regression (LR), Gradient Boosting Machine (GBM) and eXtreme Gradient Boosting (XGB)—were utilized to develop a prediction model for NM metastasis. The predictive performance of these models was evaluated using ten-fold cross-validation, radar plot and confusion matrix analysis, leading to the selection of the top-performing ML model for predictive model development. Feature importance in the optimal model was analyzed, and a network calculator was developed for model visualization.

### 4. Construction and validation of the prognostic model

Prognostic variables linked to OS in NM patients were identified through univariate Cox analysis, and variables with P < 0.05 were subsequently analyzed via multivariate Cox analysis to isolate independent prognostic factors. A nomogram was developed based on these identified independent prognostic factors. Nomogram performance was evaluated using receiver operating characteristic (ROC) curves, calibration plots, and decision curve analysis (DCA) to determine the nomogram’s clinical utility through threshold probability net benefits. Kaplan-Meier curves were generated to visualize differences in OS prognostic factors among NM patients. Patients were classified into high-risk and low-risk groups based on risk scores, with corresponding risk score maps and heat maps created for visualization.

### 5. Statistical analysis

Statistical analyses were conducted using Python (version 3.8) and R software (version 4.0.2). Count data were presented as the number of cases and percentage (%), with the Chi-squared test employed for group comparisons. The ROC curve assessed the nomogram’s discriminatory performance, the area under the curve (AUC) its predictive capacity for OS, the calibration plot its calibration, and the DCA curve its net benefit and clinical utility. The ’survivalROC’ (version 1.0.3.1), ’rms’ (version 6.3–0), ’survival’ (version 3.4–0), ’foreign’ (version 0.8–83) R packages, among others, were utilized for the construction and validation of the metastasis and prognostic model. The SHAP values for model interpretation were derived using the Python SHAP package. A P-value <0.05 was deemed to indicate statistical significance.

## Results

### 1. Patient characteristics

Data on melanoma patients from the SEER database spanning 2004 to 2017, screened according to inclusion and exclusion criteria, resulted in 4,727 eligible NM patients, comprising 2,903 (61.4%) males and 1,824 (38.6%) females, with 97.4% identified as white ethnicity (Table 1). Patients were categorized into two groups based on the occurrence of metastasis, and a chi-square test was applied to both groups. The findings indicated that 214 out of 4,727 patients exhibited metastases, with statistically significant differences (p < 0.05) observed across eight variables—gender, primary site, surgery, radiation, chemotherapy, system management, T stage and N stage—between metastatic and non-metastatic groups (Table 1).

**Table 1:** Patient baseline information.

Variables	all	No metastases	Metastases	P value
	N = 4727	N = 4513	N = 214	
**Marital** ^a^				0.012*
0	2615 (55.3%)	2494 (55.3%)	121 (56.5%)	
1	1558 (33.0%)	1477 (32.7%)	81 (37.9%)	
2	554 (11.7%)	542 (12.0%)	12 (5.61%)	
**Age** ^b^				0.403
0	1674 (35.4%)	1592 (35.3%)	82 (38.3%)	
1	3053 (64.6%)	2921 (64.7%)	132 (61.7%)	
**Race** ^c^				0.513
0	4605 (97.4%)	4396 (97.4%)	209 (97.7%)	
1	23 (0.49%)	21 (0.47%)	2 (0.93%)	
2	5 (0.11%)	5 (0.11%)	0 (0.00%)	
3	94 (1.99%)	91 (2.02%)	3 (1.40%)	
**Sequence_number** ^d^				0.848
0	2978 (63.0%)	2845 (63.0%)	133 (62.1%)	
1	1749 (37.0%)	1668 (37.0%)	81 (37.9%)	
**Gender** ^e^				<0.001*
0	2903 (61.4%)	2746 (60.8%)	157 (73.4%)	
1	1824 (38.6%)	1767 (39.2%)	57 (26.6%)	
**Laterality** ^f^				0.06
0	2016 (42.6%)	1940 (43.0%)	76 (35.5%)	
1	1908 (40.4%)	1821 (40.4%)	87 (40.7%)	
3	233 (4.93%)	219 (4.85%)	14 (6.54%)	
4	506 (10.7%)	470 (10.4%)	36 (16.8%)	
5	8 (0.17%)	8 (0.18%)	0 (0.00%)	
6	6 (0.13%)	6 (0.13%)	0 (0.00%)	
7	50 (1.06%)	49 (1.09%)	1 (0.47%)	
**Grade** ^g^				0.804
0	6 (0.13%)	6 (0.13%)	0 (0.00%)	
2	18 (0.38%)	17 (0.38%)	1 (0.47%)	
3	11 (0.23%)	11 (0.24%)	0 (0.00%)	
4	4692 (99.3%)	4479 (99.2%)	213 (99.5%)	
**Primary_Site** ^h^				<0.001*
0	1399 (29.6%)	1321 (29.3%)	78 (36.4%)	
1	1318 (27.9%)	1279 (28.3%)	39 (18.2%)	
2	791 (16.7%)	764 (16.9%)	27 (12.6%)	
3	402 (8.50%)	384 (8.51%)	18 (8.41%)	
4	184 (3.89%)	174 (3.86%)	10 (4.67%)	
5	12 (0.25%)	12 (0.27%)	0 (0.00%)	
6	63 (1.33%)	62 (1.37%)	1 (0.47%)	
7	2 (0.04%)	2 (0.04%)	0 (0.00%)	
8	546 (11.6%)	507 (11.2%)	39 (18.2%)	
9	10 (0.21%)	8 (0.18%)	2 (0.93%)	
**Surgery** ^i^				<0.001*
0	108 (2.28%)	85 (1.88%)	23 (10.7%)	
1	533 (11.3%)	449 (9.95%)	84 (39.3%)	
2	2229 (47.2%)	2171 (48.1%)	58 (27.1%)	
3	1805 (38.2%)	1757 (38.9%)	48 (22.4%)	
4	52 (1.10%)	51 (1.13%)	1 (0.47%)	
**Radiation** ^j^				<0.001*
0	4560 (96.5%)	4401 (97.5%)	159 (74.3%)	
1	167 (3.53%)	112 (2.48%)	55 (25.7%)	
**Chemotherapy** ^k^				<0.001*
0	4605 (97.4%)	4437 (98.3%)	168 (78.5%)	
1	122 (2.58%)	76 (1.68%)	46 (21.5%)	
**System_management** ^l^				<0.001*
0	4130 (87.4%)	4022 (89.1%)	108 (50.5%)	
1	597 (12.6%)	491 (10.9%)	106 (49.5%)	
**T** ^m^				<0.001*
1	508 (10.7%)	490 (10.9%)	18 (8.41%)	
2	1041 (22.0%)	1015 (22.5%)	26 (12.1%)	
3	1479 (31.3%)	1427 (31.6%)	52 (24.3%)	
4	1690 (35.8%)	1572 (34.8%)	118 (55.1%)	
5	9 (0.19%)	9 (0.20%)	0 (0.00%)	
**N** ^n^				<0.001*
0	3274 (69.3%)	3188 (70.6%)	86 (40.2%)	
1	1453 (30.7%)	1325 (29.4%)	128 (59.8%)	

^a^Marital (Married = 0, Unmarried = 1, Unknown = 2).

^b^Age (≤60 = 0, >60 = 1).

^c^Race (White = 0, Black = 1, Chinese = 2, Other = 3).

^d^Sequence number (One primary only = 0, More = 1).

^e^Gender (Male = 0, Female = 1).

^f^Laterality (Left = 0, Right = 1, Paired site, midline tumor = 3, Not a paired site = 4, Only one side, side unspecified = 5, Bilateral, single primary = 6, Unknown = 7).

^g^Grade (Well differentiated, Grade I = 0, Moderately differentiated, Grade II = 1, Poorly differentiated, Grade III = 2, Undifferentiated, anaplastic, Grade IV = 3, Unknown = 4).

^h^Primary site (Skin of trunk = 0, Skin of upper limb and shoulder = 1, Skin of lower limb and hip = 2, Skin of other and unspecified parts of face = 3, External ear = 4, Eyelid = 5, Vulva = 6, Lip = 7, Skin of scalp and neck = 8, Other = 9).

^i^Surgery (No surgery of primary site = 0, Local tumor destruction = 1, Biopsy of primary tumor followed by a gross excision of the lesion, does not have to be done under the same anesthesia = 2, Wide excision or re-excision of lesion or local amputation with margins more than 1 cm. Margins MUST be microscopically negative = 3, Other/Unknown = 4).

^j^Radiation (None/Unknown = 0, Yes = 1).

^k^Chemotherapy (None/Unknown = 0, Yes = 1).

^l^System management (None/Unknown = 0, Yes = 1, Unknown = 2).

^m^T (T1 = 1, T2 = 2, T3 = 3, T4 = 4, TX = 5).

^n^N (N0 = No regional lymph node metastasis was observed, N1 = With regional lymph node metastasis).

### 2. Model for predicting the risk of metastasis in nodular melanoma

#### 2.1 Univariate and multivariate logistic regression analysis

Univariate and multivariate logistic regression analyses were conducted on 11 patient characteristics. Univariate logistic regression analysis identified marital status (unknown, OR = 0.456, 95%CI = 0.25–0.832, p = 0.01), gender (female, OR = 0.564, 95%CI = 0.414–0.768, p < 0.001), laterality (not a paired site, OR = 1.955, 95%CI = 1.299–2.944, p = 0.001), primary site (skin of upper limb and shoulder, OR = 0.516, 95%CI = 0.349–0.764, p = 0.001; skin of lower limb and hip, OR = 0.599, 95%CI = 0.383–0.935, p = 0.024), surgery (biopsy followed by a gross excision, OR = 0.099, 95%CI = 0.058–0.168, p < 0.001; Wide excision or re-excision of lesion or local amputation with margins more than 1 cm, OR = 0.101, 95%CI = 0.059–0.174, p < 0.001; other/unknown, OR = 0.072, 95%CI = 0.009–0.553, p = 0.011), radiation (yes, OR = 13.592, 95%CI = 9.489–19.471, p < 0.001), chemotherapy (yes, OR = 15.985, 95%CI = 10.745–23.782, p < 0.001), system management (yes, OR = 8.04, 95%CI = 6.053–10.679, p < 0.001), T stage (T4, OR = 2.043, 95%CI = 1.232–3.39, p = 0.006), N stage (N1, OR = 3.581, 95%CI = 2.705–4.741, p < 0.001) as factors associated with metastasis in NM patients (Table 2).

**Table 2:** Univariate and multivariate logistic regression.

Characteristics	Category	Univariate analysis		Multivarite analysis	
		OR (95% CI)	P value	OR (95% CI)	P value
**Age**	0	Ref	Ref	Ref	Ref
	1	0.877 (0.662–1.163)	0.364	\	\
**Marital**	0	Ref	Ref	Ref	Ref
	1	1.13 (0.847–1.509)	0.405	0.996 (0.706–1.406)	0.983
	2	0.456 (0.25–0.832)	0.01*	0.344 (0.179–0.66)	0.001*
**Race**	0	Ref	Ref	Ref	Ref
	1	2.003 (0.467–8.6)	0.35	\	\
	2	0 (0-Inf)	0.977	\	\
	3	0.693 (0.218–2.209)	0.536	\	\
**Sequence.number**	0	Ref	Ref	Ref	Ref
	1	1.039 (0.783–1.378)	0.792	\	\
**Gender**	0	Ref	Ref	Ref	Ref
	1	0.564 (0.414–0.768)	<0.001*	0.591 (0.392–0.893)	0.012*
**Laterality**	0	Ref	Ref	Ref	Ref
	1	1.22 (0.891–1.67)	0.216	1.147 (0.801–1.644)	0.454
	3	1.632 (0.907–2.935)	0.102	1.117 (0.529–2.358)	0.772
	4	1.955 (1.299–2.944)	0.001*	2.089 (0.733–5.957)	0.168
	5	0 (0-Inf)	0.981	0 (0-Inf)	0.986
	6	0 (0-Inf)	0.983	0 (0-Inf)	0.988
	7	0.521 (0.071–3.823)	0.521	0.165 (0.015–1.834)	0.142
**Grade**	0	Ref	Ref	Ref	Ref
	2	338694.854 (0-Inf)	0.983	\	\
	3	1 (0-Inf)	1	\	\
	4	273814.259 (0-Inf)	0.983	\	\
**Primary_Site**	0	Ref	Ref	Ref	Ref
	1	0.516 (0.349–0.764)	0.001*	0.614 (0.384–0.98)	0.041*
	2	0.599 (0.383–0.935)	0.024*	0.607 (0.356–1.035)	0.067
	3	0.794 (0.47–1.342)	0.389	0.926 (0.502–1.709)	0.807
	4	0.973 (0.495–1.916)	0.938	1.469 (0.683–3.158)	0.325
	5	0 (0-Inf)	0.976	0 (0-Inf)	0.985
	6	0.273 (0.037–1.996)	0.201	0.138 (0.014–1.376)	0.091
	7	0 (0-Inf)	0.99	0 (0-Inf)	0.992
	8	1.303 (0.875–1.939)	0.193	0.733 (0.268–2.007)	0.546
	9	4.234 (0.884–20.275)	0.071	6.53 (0.819–52.044)	0.076
**Surgery**	0	Ref	Ref	Ref	Ref
	1	0.691 (0.413–1.159)	0.161	0.472 (0.251–0.889)	0.02
	2	0.099 (0.058–0.168)	<0.001*	0.057 (0.03–0.109)	<0.001*
	3	0.101 (0.059–0.174)	<0.001*	0.051 (0.026–0.1)	<0.001*
	4	0.072 (0.009–0.553)	0.011*	0.035 (0.004–0.336)	0.004*
**Radiation**	0	Ref	Ref	Ref	Ref
	1	13.592 (9.489–19.471)	<0.001*	5.108 (3.244–8.042)	<0.001*
**Chemotherapy**	0	Ref	Ref	Ref	Ref
	1	15.985 (10.745–23.782)	<0.001*	3.183 (1.863–5.439)	<0.001*
**System_management**	0	Ref	Ref	Ref	Ref
	1	8.04 (6.053–10.679)	<0.001*	4.536 (2.999–6.861)	<0.001*
**T**	1	Ref	Ref	Ref	Ref
	2	0.697 (0.379–1.284)	0.247	0.852 (0.425–1.708)	0.652
	3	0.992 (0.575–1.712)	0.977	0.832 (0.442–1.566)	0.569
	4	2.043 (1.232–3.39)	0.006*	1.202 (0.66–2.191)	0.548
	5	0 (0–3.78286502062044e+245)	0.969	0 (0-Inf)	0.984
**N**	0	Ref	Ref	Ref	Ref
	1	3.581 (2.705–4.741)	<0.001*	1.571 (1.091–2.262)	0.015*

OR, odds ratio; CI, confidence interval.

Subsequent multivariate logistic regression analysis determined marital status (unknown, OR = 0.344, 95%CI = 0.179–0.66, p = 0.001), gender (female, OR = 0.591, 95%CI = 0.392–0.893, p = 0.012), primary site (skin of upper limb and shoulder, OR = 0.614, 95%CI = 0.384–0.98, p = 0.041), surgery (biopsy followed by a gross excision, OR = 0.057, 95%CI = 0.03–0.109, p < 0.001; wide excision or re-excision of lesion or local amputation with margins more than 1 cm, OR = 0.051, 95%CI = 0.026–0.1, p < 0.001; other/unknown, OR = 0.035, 95%CI = 0.004–0.336, p = 0.004), radiation (yes, OR = 5.108, 95%CI = 3.244–8.042, p<0.001), chemotherapy (yes, OR = 3.183, 95%CI = 1.863–5.439, p < 0.001), system management (yes, OR = 4.536, 95%CI = 2.999–6.861, p < 0.001), and N stage (N1, OR = 1.571, 95%CI = 1.091–2.262, p = 0.015) as independent risk factors for metastasis in NM patients (Table 2).

#### 2.2 Selection and validation for the optimal machine learning model

**Fig 2:**
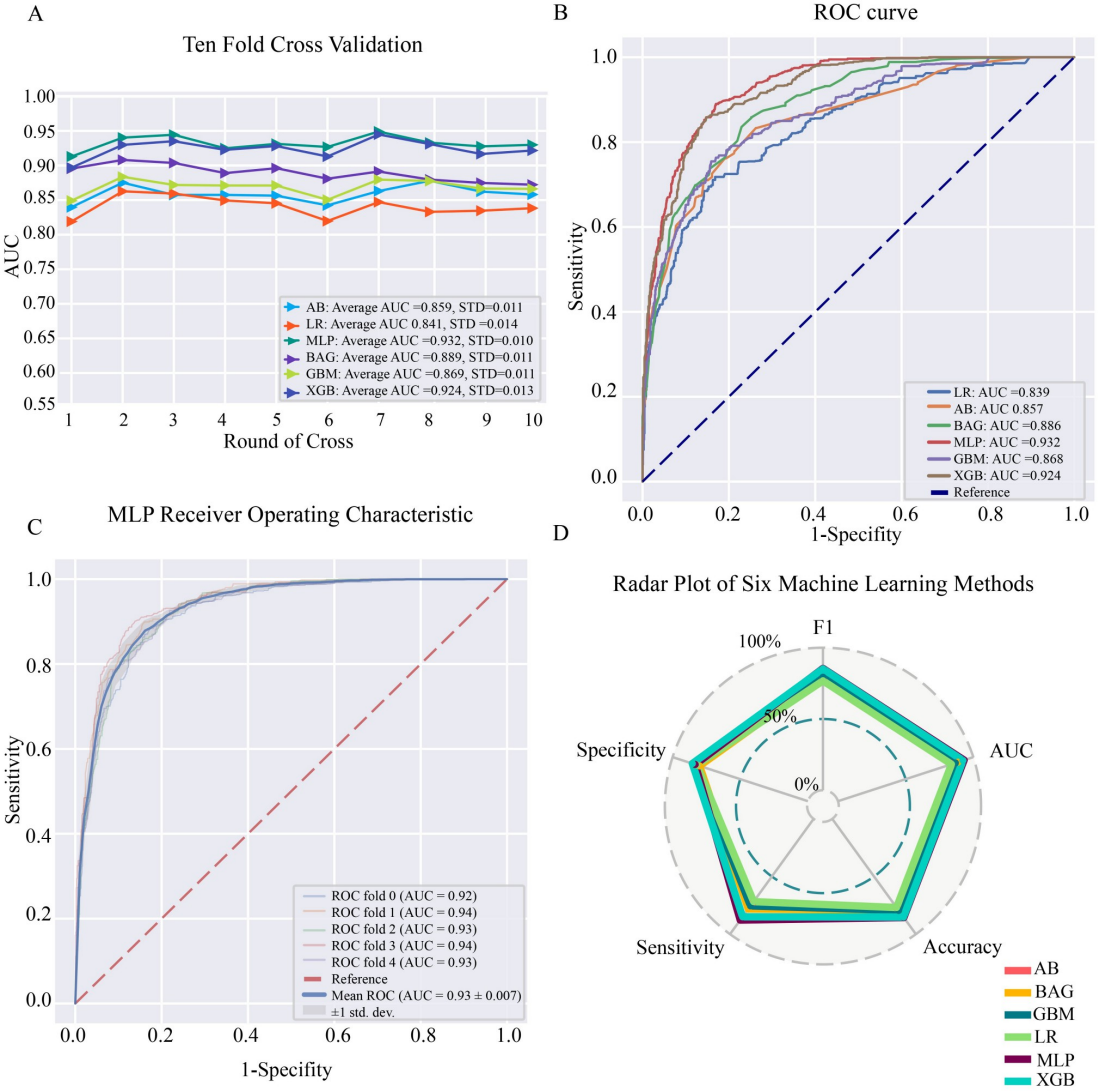
(A) Ten-fold cross-validation of the six machine learning models. (B) The ROC curves of the six machine learning models. 1-Specificity indicates the false positive rate of the model and Sensitivity indicates the true positive rate. (C) The ROC curves of the MLP. (D) The radar plots of the six machine learning models. AUC, the area under the curve; ROC, Receiver Operator Characteristic; MLP, Multilayer Perceptron; AB, Adaptive Boosting; BAG, Bagging; LR, Logistic regression; GBM, Gradient Boosting Machine; XGB, eXtreme Gradient Boosting.

Six ML algorithms—MLP, AB, BAG, LR, GBM and XGB—were utilized to develop prediction models for NM metastasis. Ten-fold cross-validation, used to compare the models’ predictive performance, revealed that all six models achieved AUC values above 0.8. MLP ranked as the most accurate, followed by XGB, with the conventional LR model as the least effective (Fig 2A). Model discrimination was assessed using the AUC of the ROC curves. The ROC analysis for the six ML models indicated strong performance and discrimination across all, with MLP outperforming the rest (Fig 2B). The MLP prediction model’s average AUC under the ROC curve was 0.93±0.007, demonstrating a high level of discrimination (Fig 2C).

**Fig 3:**
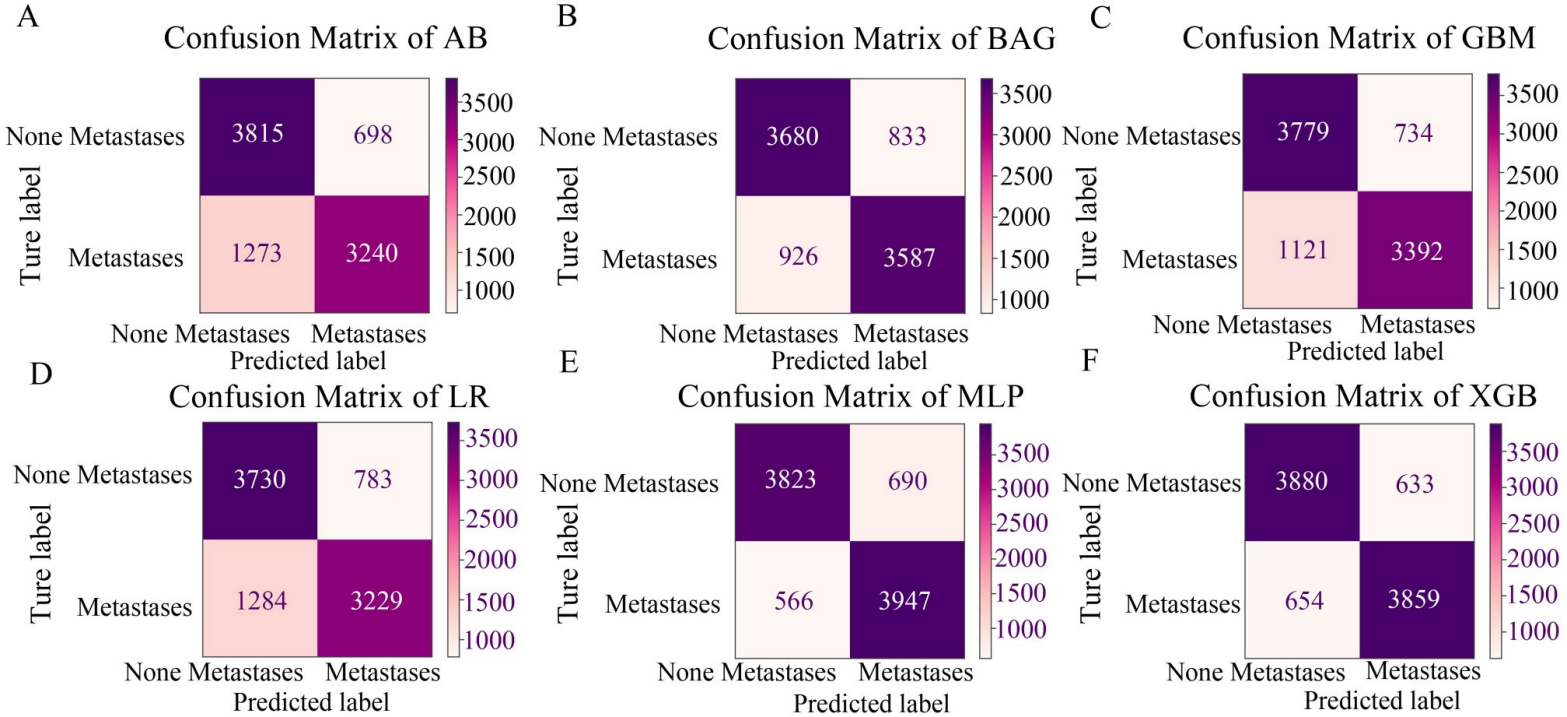
The confusion matrix for six algorithmic models. MLP, Multilayer Perceptron; AB, Adaptive Boosting; BAG, Bagging; LR, Logistic regression; GBM, Gradient Boosting Machine; XGB, eXtreme Gradient Boosting.

Furthermore, when comparing F1, Accuracy, Sensitivity, and Specificity among the six models, MLP exhibited the highest F1, Accuracy, and Sensitivity scores (F1 = 0.855, Accuracy = 0.856, Sensitivity = 0.878), showcasing its superior accuracy and sensitivity (Table 3). This underscores the model’s high accuracy and sensitivity (Table 3). Radar plots demonstrated that both the MLP and XGB models were excelled in F1, AUC, Accuracy, Sensitivity, and Specificity metrics, with MLP emerging as the favored choice (Fig 2D). Analysis of the differences between the classification outcomes of the six algorithmic models and the actual results, conducted through the confusion matrix, confirmed MLP as the optimal model (Fig 3).

**Table 3:** Comparison of six machine learning methods.

Model	F1	AUC	Accuracy	Sensitivity	Specificity
**AB**	0.781	0.857	0.782	0.726	0.839
**LR**	0.768	0.839	0.769	0.718	0.821
**BAG**	0.791	0.886	0.791	0.782	0.801
**MLP**	0.855	0.932	0.856	0.878	0.833
**GBM**	0.793	0.868	0.794	0.755	0.834
**XGB**	0.852	0.924	0.852	0.85	0.854

AB, Adaptive Boosting; LR, Logistic regression; BAG, Bagging; MLP, Multilayer Perceptron; GBM, Gradient Boosting Machine; XGB, eXtreme Gradient Boosting.

In conclusion, MLP was ultimately selected as the predictive model for NM metastasis, showcasing its superior performance across various metrics.

#### 2.3 Feature importance and visualization of the MLP model

**Fig 4:**
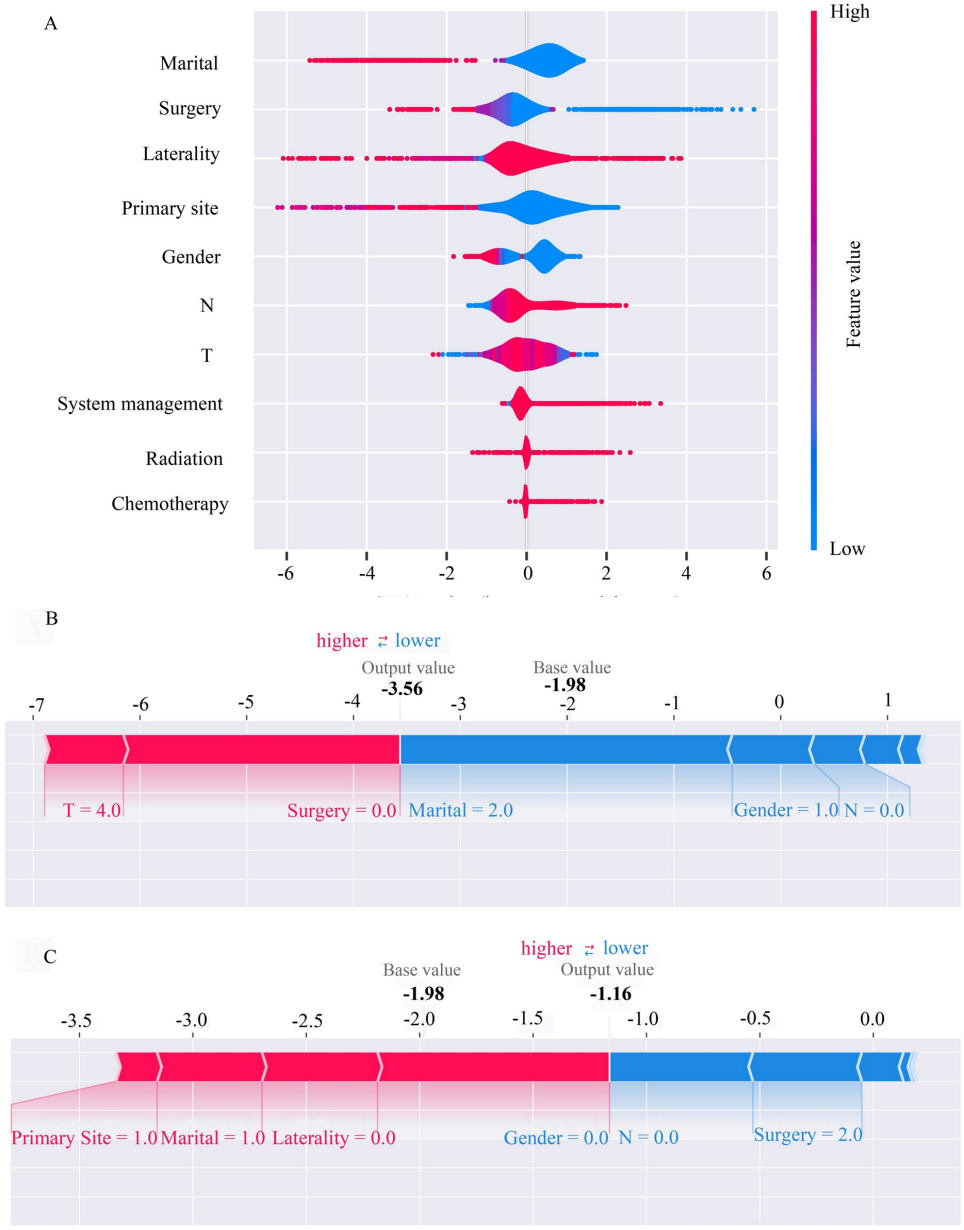
Explanation of feature importance for MLP model using SHAP. (A) SHAP summary plot. X-axis is determined by each Shapley value. (B)(C) SHAP force plot. Features predicted to increase the output are depicted in red, while those predicted to decrease the output are in blue. The length of an arrow correlates directly with the magnitude of the feature’s impact on the output. MLP, Multilayer Perceptron; SHAP, SHapley Additive exPlanation.

Feature importance of the MLP model was interpreted using the SHapley Additive exPlanation (SHAP). The marginal contribution of the ten features of the model output was interpreted for all samples, combining the feature importance and feature effects with a summary plot. The results demonstrated that the sample distribution for the features of laterality, and surgery was more dispersed, with a wide range of Shapley value, indicating a significant impact of laterality and surgery. In contrast, the distribution of chemotherapy was centered around SHAP = 0, indicating the least impact (Fig 4A). Subsequently, a force plot was created to illustrate the feature interpretation of predictions for individual sample (Fig 4B and 4C).

Concurrently, a web-based calculator was developed to visualize the model and support the clinical application of metastasis prediction in NM patients (https://shimunana-nm-distant-m-nm-m-distant-8z8k54.streamlit.app/).

### 3. The prognostic model for nodular melanoma

#### 3.1 Univariate and multivariate Cox regression

In conjunction with the established MLP metastasis model, a prognostic model for NM was further developed. Univariate and multivariate Cox regression analyses were employed to identify prognostic variables associated with survival in NM patients. Univariate Cox analysis of all variables in the sample revealed that factors such as MLP, age, marital, race, sequence number, gender, laterality, primary site, surgery, radiation, chemotherapy, system treatment, T stage, and N stage were significantly correlated with the prognosis of NM patients (p < 0.05) (Table 4).

**Table 4:** Univariate and multivariate Cox regression.

Characteristics	Category	HR (95% CI)	P value	HR (95% CI)	P value
**MLP**	\	3.337 (2.82–3.949)	<0.001*	1.61 (1.267–2.046)	<0.001*
**Age**	0	Ref	Ref	Ref	Ref
	1	2.513 (2.212–2.855)	<0.001*	2.39 (2.091–2.732)	<0.001*
**Marital**	0	Ref	Ref	Ref	Ref
	1	1.556 (1.393–1.738)	<0.001*	1.499 (1.334–1.684)	<0.001*
	2	1.191 (1.003–1.414)	0.047	1.162 (0.956–1.412)	0.131
**Race**	0	Ref	Ref	Ref	Ref
	1	2.088 (1.183–3.686)	0.011*	1.537 (0.862–2.738)	0.145
	2	0.547 (0.077–3.889)	0.547	0.62 (0.087–4.417)	0.633
	3	0.86 (0.57–1.299)	0.474	0.743 (0.49–1.128)	0.163
**Sequence.number**	0	Ref	Ref	Ref	Ref
	1	1.412 (1.272–1.568)	<0.001*	1.194 (1.072–1.33)	0.001*
**Gender**	0	Ref	Ref	Ref	Ref
	1	0.834 (0.748–0.93)	0.001*	0.928 (0.819–1.052)	0.243
**Laterality**	0	Ref	Ref	Ref	Ref
	1	0.962 (0.857–1.081)	0.518	0.985 (0.877–1.107)	0.804
	3	1.169 (0.924–1.48)	0.194	1.192 (0.924–1.539)	0.177
	4	1.275 (1.079–1.506)	0.004*	0.851 (0.593–1.222)	0.382
	5	3.749 (1.553–9.05)	0.003*	2.446 (0.991–6.038)	0.052
	6	1.934 (0.622–6.015)	0.254	2.114 (0.673–6.639)	0.2
	7	2.129 (1.416–3.202)	<0.001*	2.219 (1.451–3.393)	<0.001*
**Grade**	0	Ref	Ref	Ref	Ref
	2	3.011 (0.381–23.764)	0.296	\	\
	3	1.482 (0.154–14.244)	0.734	\	\
	4	2.23 (0.314–15.846)	0.423	\	\
**Primary_Site**	0	Ref	Ref	Ref	Ref
	1	0.919 (0.799–1.057)	0.237	0.968 (0.83–1.129)	0.677
	2	0.868 (0.736–1.024)	0.093	0.921 (0.769–1.104)	0.374
	3	1.215 (1.004–1.469)	0.045*	1.091 (0.893–1.333)	0.394
	4	1.309 (1.01–1.697)	0.042*	1.213 (0.927–1.587)	0.159
	5	0.205 (0.029–1.456)	0.113	0.301 (0.042–2.15)	0.231
	6	1.963 (1.339–2.878)	0.001*	1.782 (1.069–2.971)	0.027
	7	0 (0—Inf)	0.976	0 (0—Inf)	0.982
	8	1.224 (1.03–1.456)	0.022*	1.273 (0.9–1.801)	0.172
	9	1.296 (0.484–3.47)	0.605	0.737 (0.264–2.061)	0.561
**Surgery**	0	Ref	Ref	Ref	Ref
	1	0.815 (0.61–1.088)	0.166	1.077 (0.791–1.467)	0.637
	2	0.295 (0.224–0.39)	<0.001*	0.503 (0.368–0.687)	<0.001*
	3	0.286 (0.217–0.378)	<0.001*	0.474 (0.344–0.653)	<0.001*
	4	0.583 (0.364–0.936)	0.026*	0.625 (0.375–1.043)	0.072
**Radiation**	0	Ref	Ref	Ref	Ref
	1	2.802 (2.294–3.421)	<0.001*	1.674 (1.351–2.074)	<0.001*
**Chemotherapy**	0	Ref	Ref	Ref	Ref
	1	3.11 (2.46–3.931)	<0.001*	2.208 (1.662–2.934)	<0.001*
**System_management**	0	Ref	Ref	Ref	Ref
	1	1.279 (1.092–1.499)	0.002*	0.726 (0.596–0.884)	0.001*
**T**	1	Ref	Ref	Ref	Ref
	2	0.985 (0.768–1.263)	0.905	1.066 (0.829–1.37)	0.617
	3	1.698 (1.356–2.127)	<0.001*	1.636 (1.301–2.058)	<0.001*
	4	3.301 (2.659–4.098)	<0.001*	2.604 (2.081–3.259)	<0.001*
	5	2.522 (0.351–18.111)	0.358	2.412 (0.334–17.437)	0.383
**N**	0	Ref	Ref	Ref	Ref
	1	1.684 (1.513–1.874)	<0.001*	1.407 (1.248–1.587)	<0.001*

MLP, Multilayer Perceptron; HR, harzard ratio; CI, confidence interval.

Subsequent multivariate Cox regression analysis identified MLP (HR = 1.61, 95% CI = 1.267–2.046, p < 0.001), age (>60, HR = 2.39, 95% CI = 2.091–2.732, p < 0.001), marital status (unmarried, HR = 1.499, 95% CI = 1.334–1.684, p < 0.001), sequence number (more, HR = 1.194, 95% CI = 1.072–1.33, p = 0.001), laterality (unknown, HR = 2.219, 95% CI = 1.451–3.393, p < 0.001), surgery (Biopsy followed by a gross excision, HR = 0.503, 95% CI = 0.368–0.687, p<0.001; wide excision or re-excision of lesion or local amputation with margins more than 1 cm, HR = 0.474, 95% CI = 0.344–0.653, p < 0.001), radiation (yes, HR = 1.674, 95% CI = 1.351–2.074, p < 0.001), chemotherapy (yes, HR = 1.674, 95% CI = 1.351–2.074, p < 0.001), system treatment (yes, HR = 0.726, 95% CI = 0.596–0.884, p = 0.001), T stage (T3, HR = 1.636, 95% CI = 1.301–2.058, p < 0.001; T4, HR = 2.604, 95% CI = 2.081–3.259, p < 0.001), and N stage (N1, HR = 1.407, 95% CI = 1.248–1.587, p < 0.001) as independent prognostic variables for NM patients (p < 0.01) (Table 4).

**Fig 5:**
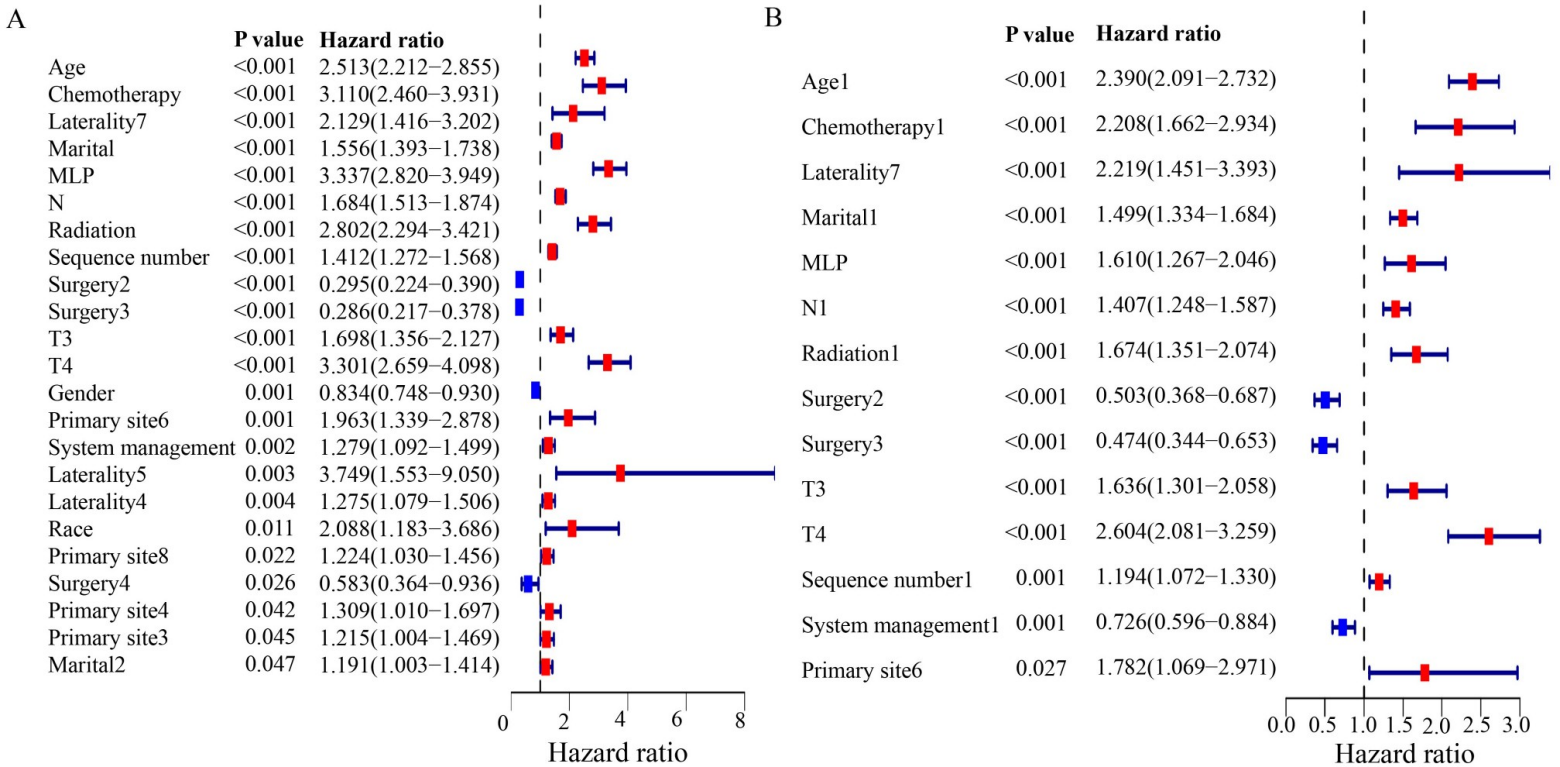
Forest plots for univariate and multivariate Cox regression.

Forest plots of univariate and multivariate Cox regression analyses revealed that system management and surgery acted as protective factors for the prognosis of NM patients (HR < 1), whereas age, chemotherapy, laterality, marital status, MLP, N stage, T stage, radiation and sequence number emerged as risk factors (HR > 1) (Fig 5).

#### 3.2 The prognostic nomogram

**Fig 6:**
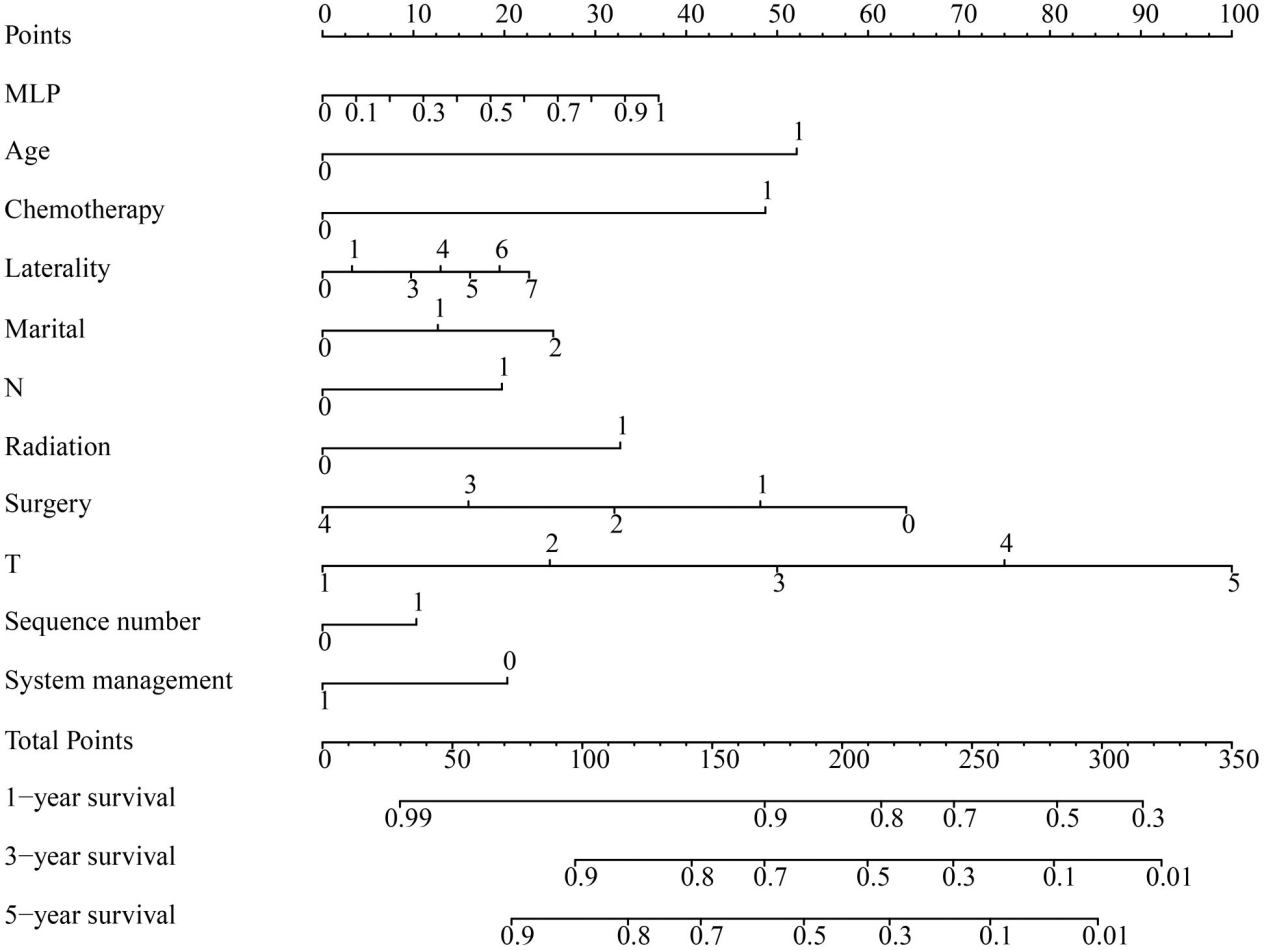
Nomogram of 1-year, 3-year, and 5-year OS for patients with nodular melanoma. Prognostic variables for a specific patient are aligned with the top "Points", generating individual scores for each variables. Subsequently, the sum of these scores is aligned with the bottom "Total Points" to estimate the patient’s overall survival probability. OS, overall survival; MLP, multilayer perceptron.

The development of a nomogram for predicting the 1-, 3- and 5-year OS in NM patients, utilizing the independent prognostic factors identified, enhances the readability of the prognostic model and offers personalized insights, aiding clinicians in evaluating patient survival and prognosis (Fig 6).

#### 3.3 Validation and evaluation of the prognostic model

**Fig 7:**
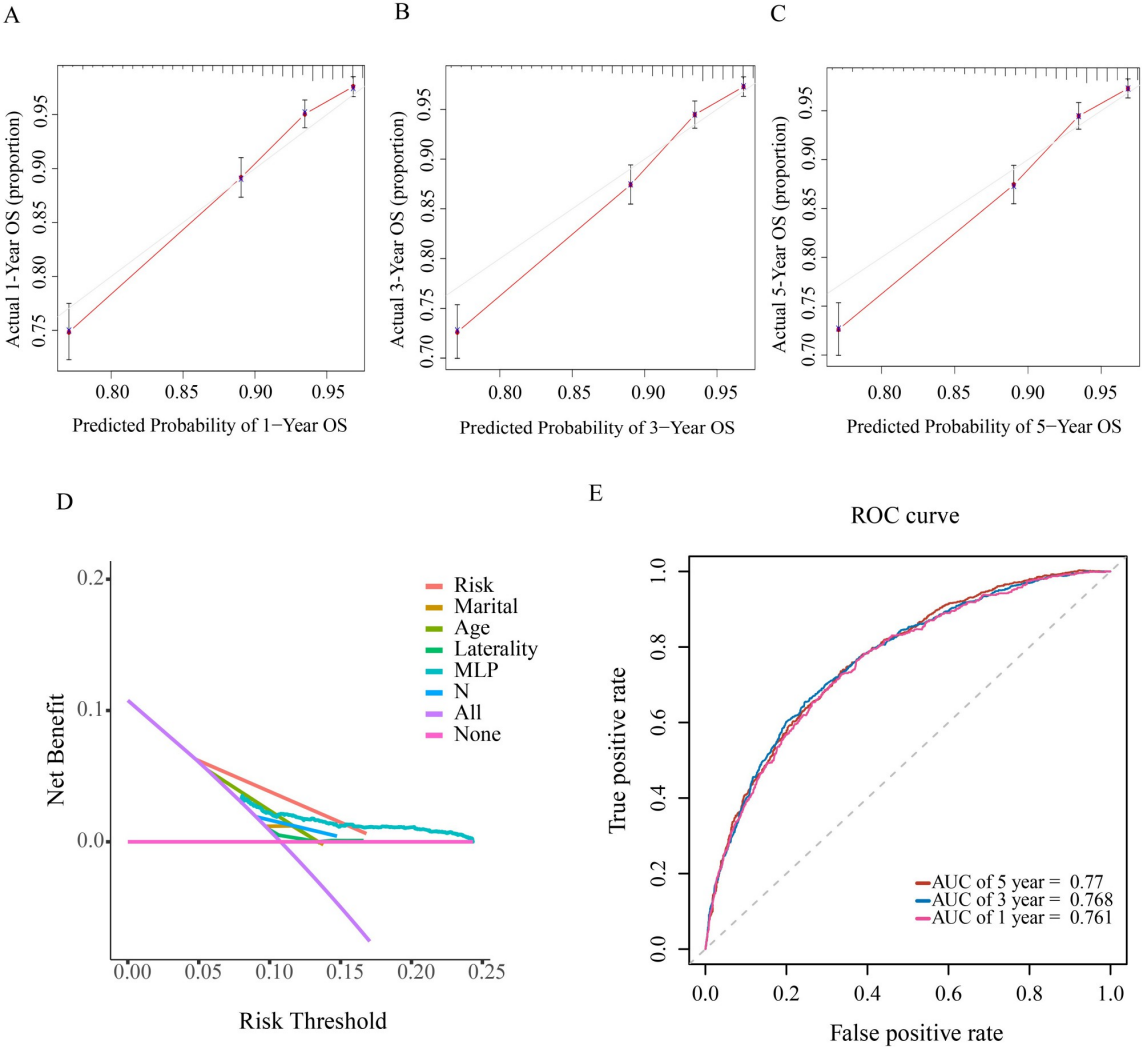
(A) (B) (C) OS calibration curves at 1, 3 and 5 years. (D) DCA curves for prognostic models. (E) ROC curves for 1-, 3- and 5- year OS. DCA, Decision Curve Analysis; ROC, Receiver Operator Characteristic.

**Fig 8:**
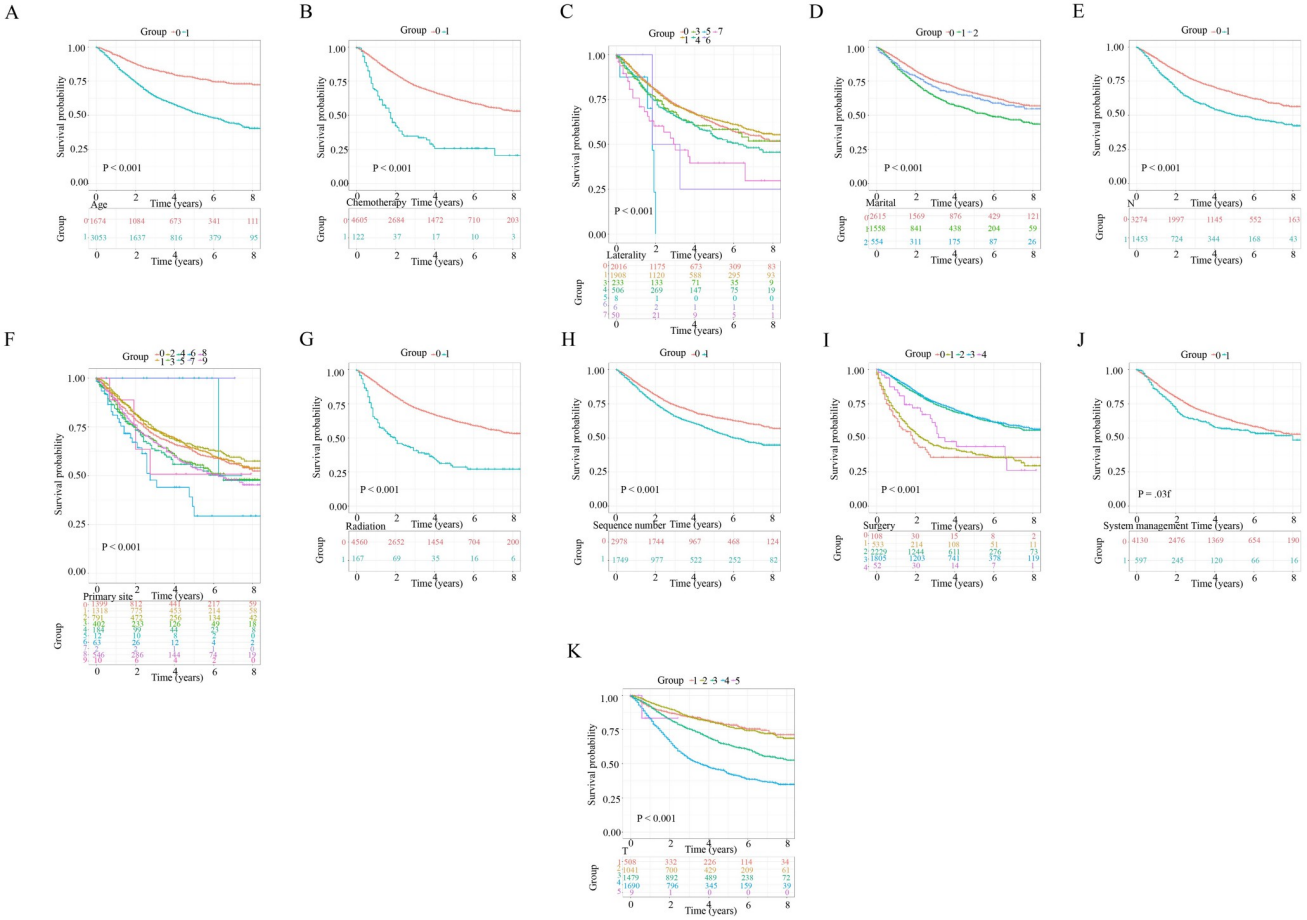
Kaplan-Meier curves for each prognostic factor in patients with nodular melanoma.

The calibration plot was employed to assess model consistency, with the 1-, 3- and 5-year OS plots closely aligning with the standard curve. This alignment indicates a strong correlation between the prognostic model and reality, demonstrating minimal discrepancy between predicted and actual prognoses (Fig 7A and 7C). The DCA curve was utilized to assess the model’s applicability, revealing that our NM prognostic nomogram exhibited significant net gain (Fig 7D), highlighting its strong clinical utility. The ROC curves evaluated the discrimination capability of the 1-year, 3-year and 5-year survival nomograms, with AUC values of 0.761, 0.768 and 0.77 respectively (Fig 7E). The Kaplan-Meier curve visualized differences in OS among NM patients’ prognostic factors. It indicated that patients over 60, with multiple tumors, unmarried, not undergoing radical surgery, with primary site in the vulva, in late stage, with lymph node metastases, or undergoing chemotherapy, systemic, and radiation therapy, had poorer prognoses (Fig 8).

**Fig 9:**
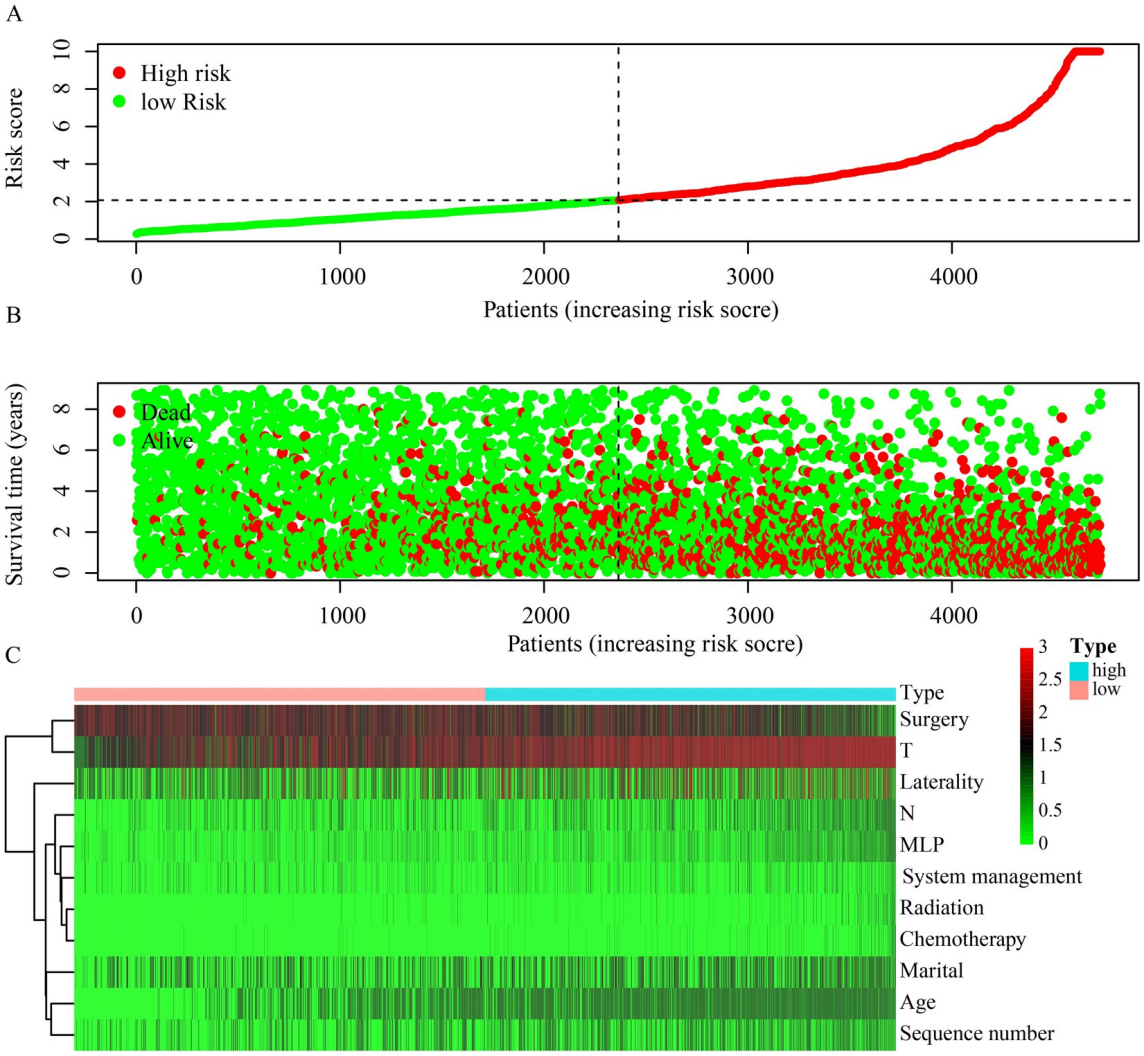
(A) Risk score grouping. (B) Risk scores and patient survival time. (C) Risk heat map. The green line represents the low-risk stratum and the red line represents the high-risk stratum.

Risk score for all samples were categorized from low to high, with the median value (risk score = 2) serving as the cutoff to classify patients into high and low-risk groups (Fig 9A). Sequentially, the risk score order from low to high revealed each patient’s survival time, with a higher mortality rate in the high-risk group compared to the low-risk group, further validating the prognostic model’s accuracy (Fig 9B). By integrating prognostic factors into a risk heat map, it was observed that high-risk patients predominantly exhibited risk factors, whereas low-risk patients showed a broader distribution of protective factors (Fig 9C).

## Discussion

NM exhibits greater aggressiveness and a higher risk of metastasis compared to other melanoma subtypes [21, 22]. Despite representing only 14% of CM [4], it constitutes a significant portion of melanomas that ultimately prove fatal [23]. Early detection and accurate diagnosis are pivotal in enhancing the prognoses. Upon diagnosis, personalized risk stratification and prognostication can extend survival by guiding the choice of optimal treatment and follow-up strategies. While the AJCC staging system is the predominant method for tumor prognosis assessment, its application to CM patient evaluation has notable limitations [24, 25]. The nomogram precisely evaluates individual survival probabilities at specified times, is user-friendly, and presents clear benefits over the AJCC staging system [26–28]. This study developed a nomogram and risk stratification system to predicting OS in NM patients, leveraging the SEER database and ML algorithms. Model validation was conducted through ROC curves, calibration plots and DCA, enhancing its predictive accuracy. The enhanced accuracy of the prognostic assessment for NM patients aids clinicians in tailoring treatment and follow-up for individual patients.

This study identified marital status, gender (female), primary site (skin of upper limb and shoulder), surgery, radiation, chemotherapy, system management, and N stage as independent risk factors for NM metastasis. Among the six ML models constructed, the MLP was selected as the predictive model for NM metastasis. Univariate and multivariate Cox analyses were conducted on the included variables to identify prognostic factors affecting OS in NM patients. Patients over 60, multiple tumors, unmarried, not undergoing radical surgery, in late stage, with lymph node metastases, or receiving chemotherapy, systemic, and radiation therapy exhibited poorer prognoses.

Generally, advanced age serves as an indicator of poorer prognosis across all histological subtypes of melanoma [29]. In this study, NM patients’ ages were categorized into two groups (≤ 60 years and > 60 years) using X-tile software. The findings indicated a deteriorating prognosis with advancing age, aligning with previous research [30]. This trend could be attributed to increased underlying disease, diminished physical function, and higher tumor burden in older patients. Hence, beyond tumor treatment, addressing underlying disease in elderly NM patients requires augmented attention. Within our patient cohort, the largest number of patients, 61.4%, were male, mirroring the recognized prevalence among NM patients [31]. Contrary to earlier findings, gender did not emerge as a risk factor for OS in NM patients in this study; however, it was identified as an independent risk factor for metastasis. Our study revealed that unmarried, older patients experienced worse prognoses. This observation is speculated to be linked to the patients’ financial circumstances and access to adequate care.

Our study identified patients with T stage T3 (depth of 2mm or more) and T4 (depth of 4mm or more) as having a poor prognosis. According to the nomogram, the T stage score holds the greatest weight for OS and is considered the most significant predictor. Distinguished from other histological subtypes, NM is characterized by a rapid vertical growth phase of invasive melanocytes, fewer radial growth phases, and absent adjacent intraepidermal spread. This vertical growth tendency leads to increased Breslow thickness in NM [32]. Studies indicate that 40% to 50% of melanomas with a Breslow thickness exceeding 2mm belong to the nodular subtype [23]. Breslow thickness serves as a crucial risk factor for NM patients [30]. This finding aligns with our results.

Lymph node metastases from NM emerged as a crucial risk factor for significantly deteriorating OS. A positive sentinel lymph node biopsy represents a significant risk factor for the substantial decline in OS [30]. Furthermore, our study revealed that NM patients undergoing chemotherapy, systemic therapy, and radiation therapy exhibited worse prognosis, potentially due to advanced stages and distant metastases.

This study detailed the primary NM occurrence sites for prognostic analysis, including the trunk, upper limbs and shoulders, lower limbs and buttocks, external ear, eyelids, vulva, lips, among others.. Within the patient cohort, the trunk was the most common site of NM, comprising 29.6% of the cases. Regarding OS, patients with NM in the vulva had the poorest prognosis. The reasons for prognostic differences in CM patients at various sites are not fully explained, but are thought to involve complex factors like regional lymphatic drainage and tumor characteristics. Given the potentially poor prognosis of patients with NM vulva origins, enhanced clinical vigilance and intensified tumor monitoring and follow-up are imperative.

Surgery remains the primary treatment modality for CM [33, 34]. Our study identified surgery as a critical prognostic factor for OS in NM patients. CM Patients undergoing surgical intervention exhibited significantly improved prognoses compared to those who did not. In early stages, most NM appears symmetrical and round, lacking specificity and potentially eluding the "ABCD" rule (asymmetry, irregular borders, color variation and diameter greater than 6 mm) [8], challenging identification even with dermoscopy. Despite the use of techniques like reflectance confocal microscopy (RCM) and optical coherence tomography (OCT) to partially improve NM detection rate, their diagnostic accuracy remains lower compared to other melanoma subtypes. To mitigate the severe consequences of NM underdiagnosis, Moscarella E et al’s study recommends the immediate removal of any nodular lesion that cannot be classified as benign [35]. While this seems a viable approach to enhancing early NM diagnosis, caution is advised in areas of functional and significant cosmetic importance to prevent excessive excision that may surpass the benefits.

While the AJCC staging system serves as a key reference for NM prognostic assessment, it includes a limited array of prognostic parameters, thus offering a restricted prognostic evaluation [36–38]. Nomogram prediction models has bridged this gap. Nevertheless, prognostic models specifically for NM patients are scarce. Based on this the current study developed a nomogram to predict OS in NM patients. Furthermore, most malignancies are classified into clinical stages based on varying risk levels in patients. Based on the risk level, appropriate adjuvant treatments or follow-up strategies are selected. In this study, we created a risk stratification for NM patients based on their nomogram scores. Our risk stratification system, containing comprehensive prognostic information, enables the identification of NM patients within high-risk and low-risk categories, accurately differentiating between various NM risk levels risk levels. This risk stratification not only underpins further prognosis but also facilitates the creation of tailored treatment plans and follow-up strategies based on patients risk levels.

Owing to database and analysis tools limitations, this study faces shortcomings that require future supplementation and compensation. First, the absence of detailed data on influencing factors, such as LDH levels, gene mutation statuses, and targeted drugs use undeniably reduces the model’s predictive accuracy. Second, the data sourced from the SEER database lack external validation, necessitating further verification of their broad applicability. Lastly, despite being based on a multicenter, large-sample database, this retrospective study harbors inherent flaws, such as the exclusion of CM patients with incomplete data, introducing selection bias. The next step include enhancing data collection on prognostic indicators for NM patients and and conducting external validation of the predictive model. A multicenter prospective study of NM will be pursued when feasible, aiming to refine prognostic assessment accuracy and support clinical decision-making. Furthermore, integrating the model with more sophisticated algorithmic approaches in future work is envisioned.

## Conclusions

This study explored risk and prognostic factors related to metastasis in NM patients, validating and assessing a ML-enhanced nomogram for predicting OS. A quantitative prognosis assessment for NM patients was achieved, offering guidance for clinical decision-making. The model is low-cost, non-invasive, and easy to implement, useful for quantifying metastasis risk in NM patients and assessing survival. It enables early identification of high-risk patients, personalized and precise treatment, and the development of follow-up strategies. The model, of course, requires further real-world validation.

## Supporting information

S1 File (DOCX)

## References

[1] Nakamura K. and Okuyama R., Changes in the Immune Cell Repertoire for the Treatment of Malignant Melanoma. Int J Mol Sci, 2022. 23(21). doi: 10.3390/ijms232112991 36361781 PMC9658693

[2] Ferrara G. and Argenziano G., The WHO 2018 Classification of Cutaneous Melanocytic Neoplasms: Suggestions From Routine Practice. Front Oncol, 2021. 11: p. 675296. doi: 10.3389/fonc.2021.675296 34277420 PMC8283700

[3] Longo C. and Pellacani G., Melanomas. Dermatol Clin, 2016. 34(4): p. 411–419. doi: 10.1016/j.det.2016.05.004 27692447

[4] Liu W., et al., Rate of growth in melanomas: characteristics and associations of rapidly growing melanomas. Arch Dermatol, 2006. 142(12): p. 1551–8. doi: 10.1001/archderm.142.12.1551 17178980

[5] Greenwald H.S., Friedman E.B., and Osman I., Superficial spreading and nodular melanoma are distinct biological entities: a challenge to the linear progression model. Melanoma Res, 2012. 22(1): p. 1–8. doi: 10.1097/CMR.0b013e32834e6aa0 22108608 PMC3253944

[6] Dessinioti C., et al., Not all melanomas are created equal: a review and call for more research into nodular melanoma. Br J Dermatol, 2021. 185(4): p. 700–710. doi: 10.1111/bjd.20388 33864261

[7] Mar V., et al., Nodular melanoma: a distinct clinical entity and the largest contributor to melanoma deaths in Victoria, Australia. J Am Acad Dermatol, 2013. 68(4): p. 568–575. doi: 10.1016/j.jaad.2012.09.047 23182058

[8] Corneli P., et al., Improving the early diagnosis of early nodular melanoma: can we do better? Expert Rev Anticancer Ther, 2018. 18(10): p. 1007–1012. doi: 10.1080/14737140.2018.1507822 30079779

[9] Wee E., et al., Clinically amelanotic or hypomelanotic melanoma: Anatomic distribution, risk factors, and survival. J Am Acad Dermatol, 2018. 79(4): p. 645–651.e4. doi: 10.1016/j.jaad.2018.04.045 30241625

[10] Lallas A., et al., Management rules to detect melanoma. Dermatology, 2013. 226(1): p. 52–60. doi: 10.1159/000346645 23485555

[11] Bunnell A.M., Nedrud S.M., and Fernandes R.P., Classification and Staging of Melanoma in the Head and Neck. Oral Maxillofac Surg Clin North Am, 2022. 34(2): p. 221–234. doi: 10.1016/j.coms.2021.12.001 35491079

[12] Gershenwald J.E., et al., Melanoma staging: Evidence-based changes in the American Joint Committee on Cancer eighth edition cancer staging manual. CA Cancer J Clin, 2017. 67(6): p. 472–492. doi: 10.3322/caac.21409 29028110 PMC5978683

[13] Mahar A.L., et al., Critical Assessment of Clinical Prognostic Tools in Melanoma. Ann Surg Oncol, 2016. 23(9): p. 2753–61. doi: 10.1245/s10434-016-5212-5 27052645

[14] Scolyer R.A., et al., Melanoma pathology reporting and staging. Mod Pathol, 2020. 33(Suppl 1): p. 15–24. doi: 10.1038/s41379-019-0402-x 31758078

[15] Kaur I., Doja M.N., and Ahmad T., Data mining and machine learning in cancer survival research: An overview and future recommendations. J Biomed Inform, 2022. 128: p. 104026. doi: 10.1016/j.jbi.2022.104026 35167976

[16] Greener J.G., et al., A guide to machine learning for biologists. Nat Rev Mol Cell Biol, 2022. 23(1): p. 40–55. doi: 10.1038/s41580-021-00407-0 34518686

[17] Deo R.C., Machine Learning in Medicine. Circulation, 2015. 132(20): p. 1920–30. doi: 10.1161/CIRCULATIONAHA.115.001593 26572668 PMC5831252

[18] Handelman G.S., et al., eDoctor: machine learning and the future of medicine. J Intern Med, 2018. 284(6): p. 603–619. doi: 10.1111/joim.12822 30102808

[19] Cui F., et al., Advancing Biosensors with Machine Learning. ACS Sens, 2020. 5(11): p. 3346–3364. doi: 10.1021/acssensors.0c01424 33185417

[20] Luengo J., et al., *Addressing data complexity for imbalanced data sets*: *analysis of SMOTE-based oversampling and evolutionary undersampling*. 2011. 15(10): p. 1909–1936.

[21] Schadendorf D., et al., Melanoma. Lancet, 2018. 392(10151): p. 971–984. doi: 10.1016/S0140-6736(18)31559-9 30238891

[22] Allais B.S., et al., Five-year survival in patients with nodular and superficial spreading melanomas in the US population. J Am Acad Dermatol, 2021. 84(4): p. 1015–1022. doi: 10.1016/j.jaad.2020.11.047 33253834

[23] Moloney F.J. and Menzies S.W., Key points in the dermoscopic diagnosis of hypomelanotic melanoma and nodular melanoma. J Dermatol, 2011. 38(1): p. 10–5. doi: 10.1111/j.1346-8138.2010.01140.x 21175750

[24] Balch C.M., et al., An evidence-based staging system for cutaneous melanoma. CA Cancer J Clin, 2004. 54(3): p. 131–49; quiz 182–4. doi: 10.3322/canjclin.54.3.131 15195788

[25] Weiss S.A., et al., Revisiting determinants of prognosis in cutaneous melanoma. Cancer, 2015. 121(23): p. 4108–23. doi: 10.1002/cncr.29634 26308244 PMC4666819

[26] Lin G., et al., A nomogram prognostic model for large cell lung cancer: analysis from the Surveillance, Epidemiology and End Results Database. Transl Lung Cancer Res, 2021. 10(2): p. 622–635. 33718009 10.21037/tlcr-19-517b PMC7947411

[27] Wang X., et al., From past to future: Bibliometric analysis of global research productivity on nomogram (2000–2021). Front Public Health, 2022. 10: p. 997713. doi: 10.3389/fpubh.2022.997713 36203677 PMC9530946

[28] Lin S., et al., Development and validation of a nomogram for predicting survival of advanced breast cancer patients in China. Breast, 2020. 53: p. 172–180. 32836201 10.1016/j.breast.2020.08.004 PMC7451432

[29] Seedor R.S. and Orloff M., Treatment of Metastatic Melanoma in the Elderly. Curr Oncol Rep, 2022. 24(7): p. 825–833. doi: 10.1007/s11912-022-01257-5 35316844

[30] Egger M.E., et al., Outcomes and prognostic factors in nodular melanomas. Surgery, 2012. 152(4): p. 652–9; discussion 659–60. doi: 10.1016/j.surg.2012.07.006 22925134

[31] Dakup P.P., Greer A.J., and Gaddameedhi S., *Let’s talk about sex*: *A biological variable in immune response against melanoma*. Pigment Cell Melanoma Res, 2022. 35(2): p. 268–279. 35076986 10.1111/pcmr.13028 PMC9305920

[32] Demierre M.F., et al., *Early detection of thick melanomas in the United States*: *beware of the nodular subtype*. Arch Dermatol, 2005. 141(6): p. 745–50. 15967921 10.1001/archderm.141.6.745

[33] Lopes J., et al., Melanoma Management: From Epidemiology to Treatment and Latest Advances. Cancers (Basel), 2022. 14(19). doi: 10.3390/cancers14194652 36230575 PMC9562203

[34] Swetter S.M., et al., Guidelines of care for the management of primary cutaneous melanoma. J Am Acad Dermatol, 2019. 80(1): p. 208–250. 30392755 10.1016/j.jaad.2018.08.055

[35] Moscarella E., et al., Performance of the "if in doubt, cut it out" rule for the management of nodular melanoma. Dermatol Pract Concept, 2017. 7(3): p. 1–5. doi: 10.5826/dpc.0703a01 29085713 PMC5661152

[36] Papageorgiou C., et al., Melanoma: Staging and Follow-Up. Dermatol Pract Concept, 2021. 11(Suppl 1): p. e2021162S. doi: 10.5826/dpc.11S1a162S 34447611 PMC8366306

[37] Tracey E.H. and Vij A., Updates in Melanoma. Dermatol Clin, 2019. 37(1): p. 73–82. doi: 10.1016/j.det.2018.08.003 30466690

[38] Ramalingam K. and Allamaneni S.S., Staging Melanoma: What’s Old and New. Surg Clin North Am, 2020. 100(1): p. 29–41. doi: 10.1016/j.suc.2019.09.007 31753114

