# Supplementary material for: Artificial intelligence-assisted metastasis and prognosis model for patients with nodular melanoma
Source: PLoS One. 2024 Aug 7;19(8):e0305468. doi: 10.1371/journal.pone.0305468 (PMC11305581; doi:10.1371/journal.pone.0305468)
Supplement: S1 File — (DOCX) [file pone.0305468.s001.docx]

**Supplementary Materials**

**Corresponding code**

Readers can also use these detailed parameter settings and corresponding code to reproduce our model with *Python software and R*. These codes can also be downloaded from the following publicly available github repositories.

(https://github.com/Wu-Shi-Nan/Nodular-Melanoma)

**The parameters used for the analysis**

random_state_new = 50

LogisticRegression(penalty="l2",dual=False,tol=1e-4, C=1.0, fit_intercept=True, intercept_scaling=1, class_weight=None, random_state=random_state_new, solver="lbfgs", max_iter=100, multi_class="auto", verbose=0, warm_start=False, n_jobs=None, l1_ratio=None)

MLPClassifier(hidden_layer_sizes=(100,), activation="relu", solver="lbfgs", alpha=0.0001, batch_size="auto", learning_rate="constant", learning_rate_init=1, power_t=0.5, max_iter=200, shuffle=True, random_state=random_state_new, tol=1e-4, verbose=False, warm_start=False, momentum=0.9, nesterovs_momentum=True, early_stopping=False, validation_fraction=0.1, beta_1=0.9, beta_2=0.999, epsilon=1e-8, n_iter_no_change=10, max_fun=15000)

xgb.XGBClassifier(n_estimators=100, max_depth=2, learning_rate=1, random_state=random_state_new)

BaggingClassifier(estimator=KNeighborsClassifier(), n_estimators=10, max_samples=0.5, max_features=0.5, bootstrap=True, bootstrap_features=False, oob_score=False, warm_start=False, n_jobs=None, random_state=random_state_new, verbose=0)

AdaBoostClassifier(estimator=None, n_estimators=10, learning_rate=1.0, algorithm="SAMME.R", random_state=random_state_new)

GradientBoostingClassifier(loss="log_loss", learning_rate=1, n_estimators=100, subsample=1.0, criterion="friedman_mse", min_samples_split=2, min_samples_leaf=1, min_weight_fraction_leaf=0.0, max_depth=1, min_impurity_decrease=0.0, init=None, random_state=random_state_new, max_features=None, verbose=0, max_leaf_nodes=None, warm_start=False, validation_fraction=0.1, n_iter_no_change=None, tol=1e-4, ccp_alpha=0.0)
